# Fatty alcohol production in *Lipomyces starkeyi* and *Yarrowia lipolytica*

**DOI:** 10.1186/s13068-016-0647-2

**Published:** 2016-10-24

**Authors:** Wei Wang, Hui Wei, Eric Knoshaug, Stefanie Van Wychen, Qi Xu, Michael E. Himmel, Min Zhang

**Affiliations:** 1 Biosciences Center, National Renewable Energy Laboratory, Golden, CO 80401 USA; 2 National Bioenergy Center, National Renewable Energy Laboratory, Golden, CO 80401 USA

**Keywords:** Metabolic engineering, Oleaginous yeasts, *Yarrowia lipolytica*, *Lipomyces starkeyi*, Fatty alcohols

## Abstract

**Background:**

Current biological pathways to produce biofuel intermediates amenable to separations and catalytic upgrading to hydrocarbon fuels are not cost effective. Previously, oleaginous yeasts have been investigated primarily for lipid production. However, yeasts store neutral lipids intracellularly making recovery difficult and expensive. In addition, once recovered from the cells, lipids are difficult to blend directly with the existing fuels without upgrading. We have, therefore, begun to investigate secreted fatty acid-derived products which can be easily recovered and upgraded to fuels.

**Results:**

In this study, we successfully demonstrate the production of fatty alcohols by the oleaginous yeasts, *Yarrowia lipolytica* and *Lipomyces starkeyi*, through expression of the fatty acyl-CoA reductase gene from *Marinobactor aquaeolei* VT8. This strategy resulted in the production of 167 and 770 mg/L of fatty alcohols in shake flask from *Y. lipolytica* and *L starkeyi*, respectively. When using a dodecane overlay during fermentation, 92 and 99% of total fatty alcohols produced by *Y. lipolytica* and *L. starkeyi*, respectively, were extracted into the dodecane phase, which compares favorably to the 3 and 50% recovered, respectively, without the dodecane layer. In both oleaginous yeasts, long chain length, saturated fatty alcohols, i.e., hexadecanol (C16:0) and octadecanol (C18:0), were predominant and accounted for more than 85% of the total fatty alcohols produced. To the best of our knowledge, this is the first report of fatty alcohol production in *L. starkeyi.*

**Conclusion:**

This work demonstrates that the oleaginous yeasts, *Y. lipolytica* and *L. starkeyi*, can serve as platform organisms for the production of fatty acid-derived biofuels and bioproducts.

## Background

Oleaginous yeasts have received increasing attention due to their ability to accumulate high intracellular content of neutral lipids, which are considered as alternatives to plant oils for biodiesel production [1]. Among these oleaginous species, *Yarrowia lipolytica* is a well-studied model organism for genetic and physiological research as well as for industrial applications [2–5]. The establishment of the genome sequence of *Y. lipolytica* strain E150 (CLIB99) [6, 7] and the development of critical genetic tools, such as transformation methods [8] and integrative expression cassettes [9–12], provide a solid platform for metabolic engineering. Compared with *Y. lipolytica*, *L. starkeyi* has been less well studied due to the lack of advanced genetic tools and generally insufficient knowledge of its cellular genetics. However, *L. starkeyi* is otherwise a very attractive host species, due to its high lipid content (~60%) [13] and ability to utilize biomass-derived sugars, including glucose, xylose, mannose, and galactose [14, 15]. These features make *L. starkeyi* a unique species for developing biofuels. With the very recent development of a transformation protocol [16], genetic engineering now enables *L. starkeyi* to serve as unique oleaginous yeast for further biofuel research.

Current research on oleaginous yeasts for biofuels applications has mainly focused on lipid production. Increasing lipid content in *Y. lipolytica* has been widely investigated [12, 17–20]. Lipids are stored intracellularly in a neutral form as triacylglycerol (TAG) and can be chemically transesterified to fatty acid methyl and ethyl esters (FAME and FAEE), which serve as biodiesels. Although TAGs are valuable renewable precursors for biodiesel production, they are not directly compatible with existing fuels. In addition, lipid extraction using solvent and upgrading are costly processes. We have, therefore, begun to investigate secreted fatty acid-derived products which can be easily recovered and further upgraded to fuels. Among the fatty acid-derived products in oleaginous yeasts, fatty alcohol is another valuable product having many applications, including detergents and cosmetics. Currently, fatty alcohols are primarily petroleum derived or derived from plants’ oils or animal fats [21, 22] and can be easily upgraded via hydrodeoxigenation processes. Fatty alcohols can also be biologically produced from fatty acyl-CoA by NAD(P)H-dependent fatty acyl-CoA reductase and are generating interest as a renewable biofuel. Microbial production of fatty alcohol was first reported in *Escherichia coli* and *Saccharomyces cerevisiae* by introducing a fatty acyl-CoA reductase (*far*) gene into the host [23–27]. In *E. coli*, fatty acid biosynthesis generates acyl–acyl carrier proteins (acyl-ACP) which is subsequently converted to fatty acid by thioesterase, then to fatty acyl-CoA by acyl-CoA ligase, and finally to fatty alcohols by FAR. Expressing *acr1* from *Acinetobacter calcoaceticus* along with expression of *tesA* and *fadD* in *E. coli* resulted in production of medium chain length fatty alcohols of 60 mg/L [25]. When *tesA* and *fadD* were co-expressed with the *far* gene, *Maqu_2220*, from *Marinobactor aquaeolei* VT8, the titer of fatty alcohol reached 422 mg/L in a shake flask and over 1.7 g/L in fed-batch fermenter [23]. Very recently, a novel strategy consisting of enhancing the acyl-CoA pool with expression of an orthogonal type I fatty acid synthase in conjunction with *tesA*, *fadD* and the *far* gene (*Maqu_2220* from *M. aquaeolei*), significantly enhanced the fatty alcohol production in *E. coli*, achieving a titer of 3.5 g/L [28]. Type I fatty acid synthesis system commonly found in mammals and fungi can be advantageous for fatty alcohol synthesis since it releases fatty acyl-CoA directly which can be used as substrate for FAR. However, despite of this advantage, the fatty alcohol titers in the engineered *S. cerevisiae*, which co-expressed the *far* gene, the fatty acid synthesis pathway genes as well as the regulatory genes in lipid metabolism, were relative low, i.e., 93.4 mg/L [24] for the mouse *far* gene expression, 84 mg/L for the bird *far* gene expression [29] and 330 mg/L for barn owl *far* gene expression in shake flasks [30]. In a recent report, expressing barn owl *far* gene in *Y. lipolytica* together with *dga1* and *fao1* knock out led to production of 636.89 mg/L intracellular and 53.32 mg/L extracellular fatty alcohols [31]. In contrast to the low lipid content in *Y. lipolytica*, some oleaginous yeasts are capable of accumulating up to 60% of lipid intracellularly, which implicates abundant metabolic flux to fatty acyl-CoA. This strong de novo synthesis of the fatty acid pathway can be attractive for fatty alcohol synthesis. Indeed, a very recently report by Fillet et al. showed substantially higher production of fatty alcohol reaching 800 mg/L from glucose in flask and over 8 g/L from sucrose in 7 L bioreactors by the oleaginous yeast *Rhodosporidium toruloides* expressing the *far* gene *Maqu_2220* [32].

**Fig. 1:**
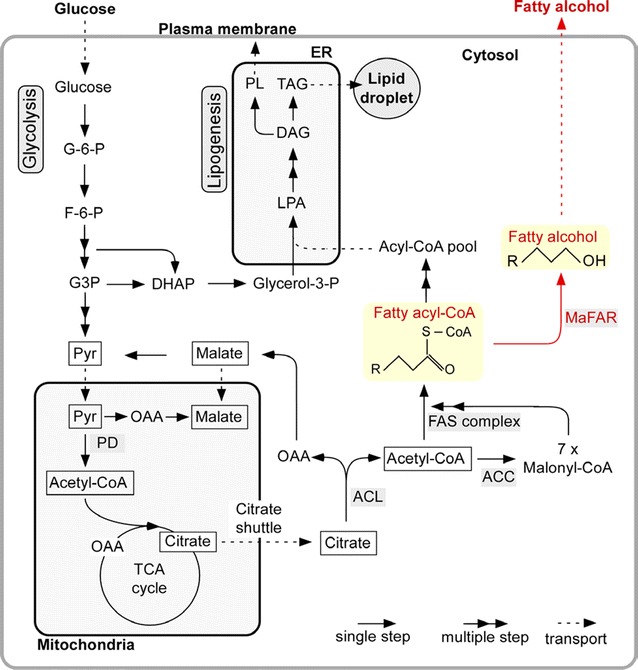
Biosynthetic pathway of fatty alcohol in *Yarrowia lipolytica* and *Lipomyces starkeyi*. The* yellow-shaded* pathway from fatty acyl-CoA to fatty alcohol indicates the engineered steps for the fatty alcohol synthesis described in this work. *ACC* acetyl-CoA carboxylase; *ACL* ATP citrate lyase; *G3P* glyceraldehyde 3-phosphate; *DGA* diacylglycerolacyltransferase; *DHAP* dihydroxyacetone phosphate; *F*-*6*-*P* fructose 6-phosphate; *FAS* fatty acid synthase; *G*-*6*-*P* glucose 6-phosphate; *LPA* lysophosphatidic acid; *MaFAR* fatty acid reductase of *Marinobactor aquaeolei* VT8; *OAA* oxaloacetate; *PD* pyruvate dehydrogenase; *PL* phospholipid; *Pyr* pyruvate; *TAG* triacylglycerol. The diagram is based on summary views of lipid biosynthesis in the recent literature described in the text of this paper

Similarly, as is the case for *R. toruloides*, *L. starkeyi* might also be a good source for fatty alcohol production considering its strong de novo synthesis of fatty acids, typically leading to ~60% intracellular neutral lipids. Although *L. starkeyi* has been genetically intractable due to lack of genetic tools until recently, a workable transformation method was introduced by Calvey et al. [16]. Compared to *Y. lipolytica*, for which genetic engineering techniques have been well established, engineering *L. starkeyi* was more challenging. In the present study, we investigated the fatty alcohol production capacity of two oleaginous yeasts, *Y. lipolytica* and *L. starkeyi*, following transformation with a heterologous *far* gene. As shown in Fig. 1 [33], fatty alcohol production is diverted from fatty acyl-CoA instead of going to TAG production . We demonstrated that the *far* gene, *Maqu_2220* from *M. aquaeolei* VT8, was successfully expressed in both *Y. lipolytica* and *L. starkeyi* and enabled the production of fatty alcohols.

## Methods

### Microorganisms and vectors

*Yarrowia lipolytica* Po1g (MatA, leu2-270, ura3-302:URA3, xpr2-332, and axp-2) and the vector pYLEX1 were purchased from Yeastern Biotech Co. (Taipei, Taiwan). The vector pMT015-YTEFin was constructed by replacing the hp4d promoter of pYLEX1 with the translation elongation factor-1a (TEF) promoter as described in the literature [18]. This promoter was synthesized (Genscript) as a* Sal*I-*Kpn*I fragment and ligated into the appropriately restricted pYLEX1 vector. The gene encoding *Maqu_2220*, a fatty acyl-CoA reductase (MaFAR) from *M. aquaeolei* VT8 [21 (Accession: YP_959486), was codon optimized and synthesized (GenScript). The synthesized gene product was ligated with the *Sna*BI–*Nsi*I double-restricted vector pMT015-YTEFin. This vector also contains a leucine selection marker gene (LEU2) to complement the deletion of the *LEU2* gene in the parent strain of Po1g. The resulting plasmid, pYLEX14, was digested with *Not*I enabling the linearized plasmid to integrate at the PBR docking platform established in the genome of *Y. lipolytica* Po1g (YLEX expression kit).

*Lipomyces starkeyi* NRRL Y-11557 used in this study was obtained from National Center for Agricultural Utilization Research, USDA-ARS, Peoria, IL, USA. Genomic DNA was isolated using the Ultraclean Soil DNA Isolation Kit (MoBio Laboratories Inc.). To transform *L. starkeyi*, a selection plasmid was constructed using the native TDH3 promoter (primers listed in Table 1. EK9, EK10) and PGK1 terminator (EK13, EK14) of *L. starkeyi* to express the NAT1 gene of *Streptomyces noursei* encoding resistance to nourseothricin. The promoter and terminator were PCR-amplified from genomic DNA having a 5′ and 3′ regions homologous to a region of pUC19 just upstream of the multicloning site. The SnNAT1 gene (EK11, EK12) was PCR-amplified from pYL16 (Werner Bioagents, Germany) having 5′ and 3′ regions of homologous overlap with the promoter and terminator, respectively, to construct the LsTDH3p-MaFAR-LsPGK1t expression cassette. The plasmid pUC19 (EK15, EK16) was also PCR-amplified with 5′ and 3′ regions of homology to the *L. starkeyi* promoter and terminators. The PCR products were gel isolated, purified using the Qiaquick Gel Extraction Kit (Qiagen), and assembled as a 4-fragment Gibson assembly reaction (Gibson Assembly Cloning Kit, NEB). This selection vector was designated pEKLs1 and was PCR-amplified (EK23, EK24) for the next construction step. The native PYK1 promoter (EK17, EK21) and GAL1 terminator (EK18, EK22) of *L. starkeyi* were PCR-amplified from genomic DNA having 5′ and 3′ regions of homology to pEKLs1 upstream of the NAT1 expression cassette The promoter, terminator, and pEKLs1 vector PCR products were gel isolated and used for a three-fragment Gibson assembly. The resulting vector was designated pEKLs2 and allows for the insertion of any gene of interest for overexpression in *L. starkeyi* by Gibson assembly between the LsPYK1p and LsGAL1t. The gene encoding MaFAR was PCR-amplified from pYLEX14 (described above) having 5′ and 3′ regions of homology to the LsPYK1p and LsGAL1t. The PCR-amplified MaFAR gene and pEKLs2 vector (EK17, EK18) were gel isolated and used for a two-fragment Gibson assembly. Correct insertion of the MaFAR gene was confirmed by DNA sequencing, and the resulting plasmid was named pLS101-4.

**Table 1:** PCR primers used in this study

EK9	AGTGAATTCCCTCTGGTACGTAAGATTACGGA
EK10	TCGTCAAGAGTGGTCATTGCGAATGTGGATTAGAGTAAGA
EK11	AATCCACATTCGCAATGACCACTCTTGACGACACGGCTTA
EK12	ACATTAACGGGAGTCAGGGGCAGGGCATGCTCATGTA
EK13	TGCCCTGCCCCTGACTCCCGTTAATGTTGGGATTCT
EK14	GAGCTCCCTGTCAATTATGCTACCACTTGGT
EK15	TAGCATAATTGACAGGGAGCTCGGTACCCGGGGATCCT
EK16	TCTTACGTACCAGAGGGAATTCACTGGCCGTCGTTT
EK17	CATGTTGGCTGTAGTGATACGGACGCA
EK18	AGTTTAGAGATGTACAAGGGGTAT
EK21	TTGTAAAACGACGGCCCGCACCTGCTGAATGCGCTGACGAT
EK22	CCAGAGGGAATTCACTGCCACGATAACTTTGTGCAAAGATA
EK23	AGTGAATTCCCTCTGGTACGTAAGATTACGGA
EK24	GGCCGTCGTTTTACAACGTCGTGACTGGGAA
515_FAR1_F2	TCCTTCAACCACTCTGCGTCCGTATCACTACAGCCAACATGGCCATTCAGCAGGTCCAC
516_FAR1_R2	TGACAATGCACCTCAATACCCCTTGTACATCTCTAAACTTCAGGCGGCCTTCTTTCGCTG
Maqu_2220F	TCGAGAGAAGGTCACCCTCT
Maqu_2220R	CCTTAGACTCGGCCATGAGG

### Transformation and selection

Targeted integrative transformation of *Y. lipolytica* with pYLEX14 was conducted as described by Wei et al. [34] using the *YLOS* One-step Transformation system included in the *YLEX* expression kit. For the selection of prototrophic recombinants (Leu^+^), transformants were grown on solid YNB medium (2% glucose w/v, 0.67% yeast nitrogen base w/o amino acids, 1.5% agar), incubated at 28 °C for 2–4 days.

Random integrative transformation of *L. starkeyi* was conducted as described by Calvey et al. [16]. A single colony of *L. starkeyi* was inoculated into YPD medium and incubated at 30 °C and shaken at 225 rpm until OD_600_ reached 5.2. Cells were harvested by centrifugation at 3000 rpm for 5 min, washed with 25 mL of water and resuspended in 0.5 mL of 0.1 M LiAc. Suspended cells (100 µL) were dispensed into a microcenrifuge tubes and briefly pulsed to collect the cells and remove the LiAc. The collected cells were resuspended in 240 µL of PEG (50% w/v, MW 3650), 30 µL of 1 M LiAc, 3 µL of ssDNA (10 µg/µL), and linearized DNA and water to equal 27 µL (300 µL total volume) and vortexed thoroughly. The mixture was incubated at 30 °C for 3 h without shaking and then treated under a 5-min thermal shock at 40 °C. The cells were pelleted by centrifugation for 15 s, and the supernatant was removed. The cell pellet was resuspended in one mL of YPD medium and incubated at 30 °C and 225 rpm for 3 h. Cells were then centrifuged, resuspended in 0.5 mL of water, and plated on YPD with 30 µg/mL of nourseothricin (ClonNAT). Transformants appeared after 5 days incubation at 30 °C. The successful transformation and integration of the *Maqu_2220* gene in both *Y. lipolytica* and *L. starkeyi* were verified by PCR amplification (see Table 1 for primer sequences) of a 206 bp fragment internal to the *Maqu_2220* gene using extracted genomic DNA as a template.

### Culturing of *far* transformants for fatty alcohol production

Cultures of *Y. lipolytica* and *L. starkeyi far* transformants were grown in 250 mL baffled shake flask. For both strains, seed cultures were first prepared in 5 mL of YPD broth in 125 mL flasks. For *Y. lipolytica*, the seed culture was incubated at 28 °C and 220 rpm. After 24 h, 2.5 mL of seed culture was inoculated into 25 mL of production medium in a 250 mL shake flask and incubated in a shaker at 28 °C and 220 rpm. For *L. starkeyi,* the seed culture was incubated at 30 °C and 220 rpm. *L. starkeyi* grew slower compared to *Yarrowia*, thus we harvested the seed culture of *Lipomyces* at 36 h instead of 24 h. The production medium inoculated with the seed culture was cultivated at 30 °C and 220 rpm. For the culture with dodecane overlay, 3 mL of dodecane was added to the production medium, 48 h after the cultivation in 250-mL flask was started. For the analysis of fatty alcohol production, cultures were harvested at 5 days unless otherwise stated and centrifuged at 4000 rpm. The cell pellet, supernatant and dodecane phase (if overlaid with dodecane) were separated after centrifugation. Flask experiments were run in triplicates, the data were shown as the average of the triplicates, and error bars represent the standard deviation of the triplicates.

The medium used for fatty alcohol production in this study was mineral medium containing (g/L): (NH_4_)_2_SO_4_, 0.5; KH_2_PO_4_, 7.0; Na_2_HPO_4_, 2.0; MgSO_4_·7H_2_O, 1.5; CaCl_2_·2H_2_O, 0.1; FeCl_3_·6H_2_O, 0.08; ZnSO_4_·7H_2_O, 0.001; CuSO_4_·5H_2_O, 0.0001; CoCL_2_·H_2_O, 0.0001; MnSO_4_· 5H_2_O, 0.0001 and vitamin supplements. The carbon source was glucose (3% w/v). The initial pH value of the medium was pH 6.0. For culturing of the control strain of *Y. lipolytica* Po1g in mineral medium, leucine was supplemented to the medium at a concentration of 0.1 g/L.

### GC–MS analysis for fatty alcohols

Fatty alcohols were extracted from media, cell pellets, and whole culture (media + pellet) using an extraction procedure similar to Steen et al. [25] for analysis (except for the culture with dodecane overlay where samples of dodecane phase were directly used for GC–MS analysis). In brief, 10 μL of a recovery standard consisting of 5 mg/mL tridecanol in ethyl acetate was spiked into 10 mL of sample and vortexed thoroughly. Spiked samples were extracted with 40% (v/v) ethyl acetate. To facilitate extraction, samples were hand shaken for 2 min before centrifugation at 4 °C for 10 min at 3000 rpm. A blank sample consisting of 10 mL of water spiked with recovery standard was also run with each extraction set. If the two phases formed an emulsion, samples were placed in a freezer (0 °C) overnight before continuing. The ethyl acetate phase was recovered and dried down under a steady stream of N_2_ to a little less than 1 mL before being quantitatively transferred to a glass GC vial, and finally brought to a 1 mL total volume.

Analysis for fatty alcohols was performed on a 7890N Agilent GC system with an Agilent DB-Wax column, 30 m × 0.25 mm × 0.25 μm. The GC parameters used were as follows: 1 μL injection, split ratio 10:1, injector temp 250 °C, constant flow 1 mL/min He; oven ramp 100 °C for 1 min, 25 °C/min to 200 °C and hold for 1 min, 5 °C/min to 250 °C and hold for 7 min; FID detector at 280 °C, 40 mL/min H_2_, 450 mL/min zero air, 30 mL/min makeup He, collection at 5 Hz. Saturated and unsaturated fatty alcohols were quantified using two fatty alcohol standards (NuChek GLC 32C and 34C). A similar program was run on the GC–MS to verify peak identities.

Where applicable, samples were overlain with 10% (v/v) dodecane during growth. The dodecane layer was recovered and diluted as necessary before being run on the GC-FID as described above. All the samples were analyzed in triplicates, and the standard deviations were <0.35%.

### Lipid determination

The cell biomass was harvested by centrifugation and freeze dried for lipid determination. 0.2 mL chloroform–methanol (2:1 v/v) was added to 7–11 mg of lyophilized biomass to solubilize the lipids and simultaneously transesterify the lipids in situ with 0.3 mL HCl–methanol (95% v/v) for 1 h at 85 °C. The resulting FAMEs were extracted with 1 mL hexane at room temperature for 1 h and analyzed by gas chromatography GC-FID (Agilent 6890N) using DB5-MS column (Agilent, USA). Quantification of the FAMEs was based on integration of individual fatty acid peaks in the chromatograms and quantified using a 5-point calibration curve of a mixed, even carbon chain FAME standard of 14 individual fatty acids (C8–C24, SIGMA cat# 18918). The total lipid content was calculated as the sum of the even numbered FAMEs contributions. All the samples were analyzed in triplicates, and the standard deviations were <0.35%.

## Results and discussion

### Production of fatty alcohols in *far*-expressing *Y. lipolytica* Yl[FAR]

**Fig. 2:**
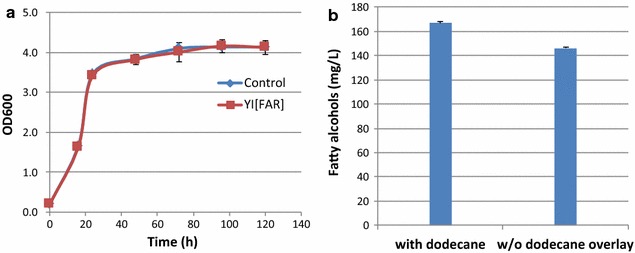
Cell growth (**a**) and fatty alcohols production (**b**) by *far*-expressing *Y. lipolytica* growing on mineral medium. The* control* represents empty vector control

A well-known and commercially available *YLEX* expression kit was used for *Y. lipolytica* transformation. The successful transformation and integration of the *Maqu_2220* gene in *Y. lipolytica* was verified by PCR amplification as described in ''Methods'' section. Due to the targeted integration at a specific docking site in *Y. lipolytica*, effects on expression due to random integration localization are not expected. Three *far* transformants were cultured and characterized in terms of cell growth and fatty alcohol production, and they turned to show very similar growth and fatty alcohol production profile. Thus only one transformant was chosen for further study. Expression of the *far* gene in *Y. lipolytica* did not impact cell growth (Fig. 2a) and resulted in the production of up to 167 mg/L of fatty alcohols in mineral medium while the control *Y. lipolytica* Po1g did not produce any fatty alcohols. This titer was approximately three times higher than that observed with the single mouse *mfar1* gene expression in *S. cerevisiae* (56.5 mg/L) [24] and six times higher than the bird *far* gene expression in *S. cerevisiae* (26 mg/L) [29]. Analyzing the cell pellets and supernatant separately showed that 97% of the produced fatty alcohol was associated with or trapped in the cells while only 3% was secreted (Table 2). This result is consistent with the recent research about *Y. lipolytica* that most of the fatty alcohols were retained intracellularly when TaFAR1 gene was expressed in *Y. lipolytica* [31]. Using a 10% (v/v) dodecane overlay during growth, 92.5% of the produced fatty alcohols were extracted into the dodecane phase, while only 5% remained associated with cells (Table 2). This high extraction efficiency by dodecane would undoubtedly benefit downstream separation processes.

**Table 2:** Fatty alcohols distribution in *Y. lipolytica* fermentation broth samples with and without dodecane overlay

Sample	Sample volume (ml)	Fatty alcohols in dodecane (mg)	Fatty alcohols in supernatant (mg)	Fatty alcohols in pellet (mg)	Total fatty alcohols (mg)
Without dodecane	55.0	N/A	0.24 ± 0.04	7.78 ± 0.10	8.02 ± 0.10
With dodecane	55.0	8.52 ± 0.02	0.23 ± 0.02	0.46 ± 0.02	9.21 ± 0.05

In this study, we also compared fatty alcohols production titers with and without dodecane overlays during the incubation. Our characterizations show that 14% more fatty alcohols were detected in dodecane-overlaid cultures than without dodecane overlays (Fig. 2b). This may be due to the inhibition or toxicity of fatty alcohols to the cells in non dodecane-overlaid cultures. We also found differences in the concentrations of fatty alcohols between the whole culture analysis and the pellet plus supernatant determined separately. For example, for the same culture, we found 21% less fatty alcohols from the whole culture analysis compared to the separate analysis of cells and supernatant (Table 3). This lower value in the measured fatty alcohol titer by the whole culture analysis method could be due to a lower extraction efficiency when cells and medium were mixed together. Overlaying dodecane appears to be the most efficient method to recover the produced fatty alcohols, thus in the following experiments, we overlaid dodecane on all the culture broths, and fatty alcohols in dodecane phase were measured.

**Table 3:** Fatty alcohols in *L. starkeyi* fractions: analyzing whole culture or analyzing cells and supernatant separately

	Fatty alcohols in pellets (mg/L)	Fatty alcohols in supernatant (mg/L)	Total fatty alcohols (mg/L)
Separate fractions	140.8 ± 2.5	4.2 ± 0.2	145.0 ± 2.2
Whole culture	N/A	N/A	115.0 ± 5.0

### Lipid accumulation and fatty alcohol composition in Yl[FAR]

**Fig. 3:**
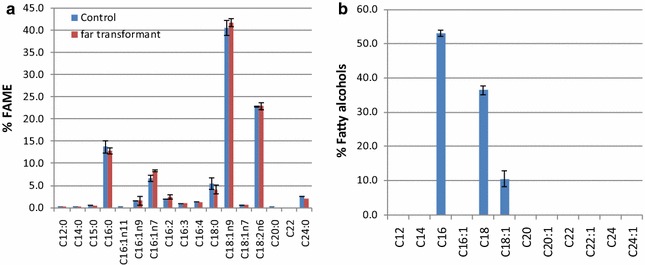
Lipid and fatty alcohol compositions in Yl[FAR] growing on mineral medium at 5 days. Fermentation medium was overlaid with dodecane. **a** Lipid composition. **b** Fatty alcohol composition. The* control* represents empty vector control

Overexpressing the *far* gene in *Y. lipolytica* led to 15% decrease in lipid content (7.9 ± 0.1% compared to the parent strain 9.3 ± 0.3%), indicating that the carbon flux originally going to TAG synthesis might partially be redirected toward fatty alcohol production. Note that the lipid composition profile in the *far* transformant was identical to that of the parent strain *Y. lipolytica* Po1g, and the lipid profile of the parent strain is consistent with the previous studies [18], i.e., C16:0 and C18:1 are two major fatty acids species (see Fig. 3a).

The composition of the fatty alcohols produced in Yl[FAR] is shown in Fig. 3b. The fatty alcohols with a long chain length, i.e., hexadecanol (C16:0), octadecanols (C18:0), and (C18:1) accounted for 53.1, 36.3, and 10.4%, respectively, of the total alcohols. It is noteworthy that saturated fatty alcohols were predominant in all the produced fatty alcohols, which could be beneficial for downstream upgrading to hydrocarbons as less hydrogen is needed in the hydrotreating process. The fatty alcohol composition profile of MaFAR transformant in this study is very similar to the recently published research on fatty alcohol production in *Y. lipolytica*, although a different source of *far* gene was expressed. Does this indicate that fatty alcohol composition is species specific? More cases of studies on different *far* gene expressions in *Y. lipolytica* will help to answer this question.

### Production of fatty alcohols in *far*-expressing *L. starkeyi* Ls[FAR]

Oleaginous yeasts require fatty acyl-CoA for de novo biosynthesis of TAG. Fatty acyl-CoA is a central intermediate in fatty acid synthesis which can be converted to various fatty acid-derived products including TAG and fatty alcohols via different enzymes. Overexpressing the *far* gene in oleaginous yeast is a straight forward strategy to bypass the TAG synthesis pathway by pulling fatty acids (in fatty acyl-CoA form) toward fatty alcohols production. The lipid content in the parent strain *Y. lipolytica* Po1g, used in this study, was less than 15% percent, whereas lipid content in *L. starkeyi* was greater than 50%. As *L. starkeyi* accumulates higher levels of TAG, we assume higher levels of fatty acyl-CoA are produced as an intermediate in *L. starkeyi* than in *Y. lipolytica*. Therefore, more fatty acyl-CoA should be available for conversion to fatty alcohols when overexpressing the *far* gene. To this end, the same MaFAR gene was also expressed in *L. starkeyi* NRRL Y-11557.

**Fig. 4:**
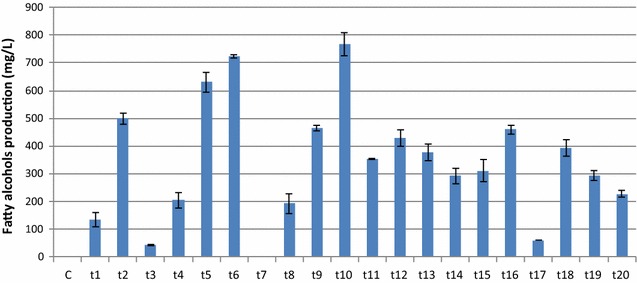
Fatty alcohol production by *far*-expressing *L. starkeyi* transformants on mineral medium at 5 days. Fermentation medium was overlaid with dodecane. *C* represents empty vector control

The successful transformation and integration of the *Maqu_2220* gene in *L. starkeyi* was verified by PCR amplification as described in ''Methods'' section. Due to the nature of random integration and the possibility of effects on gene expression due to integration localization, 20 transformants with random insertions of the *far* gene were chosen for characterization. All the transformants were confirmed to produce fatty alcohols, albeit at varied titers (Fig. 4). Considering the random insertion of *far* gene, this variation in fatty alcohols production among the transformants is not unexpected and could be due to different copy numbers or locations of the inserted *far* gene in the genome. Further work will be done to understand the mechanism involved. Among the 20 transformants found, transformants 6 and 10 produced more fatty alcohols than the others in mineral medium, 720 and 770 mg/L after 5 days, respectively. This production titer is comparable to the same *far* gene expression in *R. toruloides* grown on glucose medium. The higher level of fatty alcohol production may be related to the robust fatty acid synthesis in *L. starkeyi* as indicated by high lipid content in wild type cells. Moreover, the level of the fatty alcohols produced in *L. starkeyi* from mineral medium containing 3% glucose is very comparable to the fatty alcohol production by another high lipid-accumulating oleaginous yeast *R. toruloides*, 0.8 g/L fatty alcohols produced from YP medium containing 4% glucose [32].

### Lipid accumulation and fatty alcohol composition in *far*-expressing *Lipomyces* Ls[FAR]

**Fig. 5:**
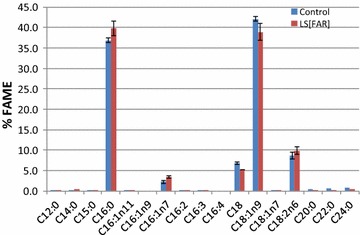
Lipid compositions in Ls[FAR] transformant 10 growing on mineral medium at 5 days. The standard deviation of triplicates was <0.35%. The control represents empty vector control

The FAME composition data of the wild type *L. starkeyi* in our study were consistent with the previous studies on this strain, i.e., C16:0 and C18:1 fatty acids were predominant [13]. Similar to *Y. lipolytica*, expression of the *far* gene in *L. starkeyi* also led to a decrease in lipid content while lipid composition profiles remained the same as the parental control (Fig. 5). Considering transformant 10, for example, FAME content was found to decrease to 40.6% at 5 days, compared to 56.9% in the control.

Although fatty alcohols were produced by expressing the *far* gene in *L. starkeyi*, we found that there remained a large amount of lipid accumulation in the cells, even though we tried to bypass the TAG synthesis pathway by redirecting carbon flux to fatty alcohols production. A much stronger “pull” enhancing MaFAR expression is needed. In addition to the “pull” strategy, blocking TAG synthesis, i.e., disruption of the *dga* gene which encodes for diacylglycerol acyltransferase, the final enzyme for TAG synthesis, is another strategy for further enhancing fatty alcohol production. Also, as demonstrated in recent research [31], eliminating fatty alcohol degradation pathway, might be another strategy deserving a try. However, considering the complex nature of this species and for other unknown genetic reasons, the subsequent knocking out genes is very challenging. To this end, a more efficient transformation system able to achieve targeted gene knock-out must be developed.

**Fig. 6:**
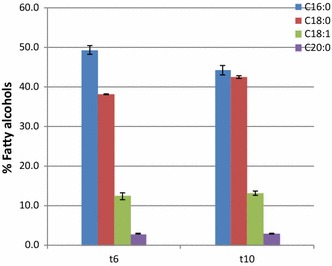
Fatty alcohol compositions in Ls[FAR] transformants 6 and 10 growing on mineral medium at 5 days

The patterns of fatty alcohols composition in *L. starkeyi far* transformants were similar to that of *Y. lipolytica*. In all the *far* transformants, long chain fatty alcohols i.e., saturated hexadecanol (C16:0) and octadecanol (C18:0) accounted for 85–88% of the total fatty alcohols, whereas unsaturated octadecanol (C18:1) accounted for 12–15%. This result is in contrast to the fatty alcohols composition in *R. toruloides*, which showed a very high content of unsaturated oleyl alcohol (C18:1, 69%) and low contents of saturated hexadecanol (C16:0) and octadecanol (C18:0) (12% and 19%, respectively) [32]. In transformants 6 and 10, the two *far* transformants with the highest fatty alcohols titers, a small fraction (about 2.5%) of long chain fatty alcohol (C20:0) was also observed (Fig. 6).

The fatty alcohols profiles in *Y. lipolytica* and *L. starkeyi* are pretty similar showing that saturated C16:0 and C18:0 fatty alcohols predominate, accounting for 85–90% of total fatty alcohols. It is noteworthy that this is different from the composition profiles of *R. toruloides* and *E. coli* ever reported, although the same MaFAR was expressed [23, 32]. In *R. toruloides*, unsaturated oleyl alcohol (C18:1) was predominant, accounting for 57% of the total fatty alcohols [32]. In *E. coli*, C16:0 and C18:1 were major components, but about 15% of shorter chain length C12:0 and C14:0 fatty alcohols also existed [23]. This indicates that the *far* gene *Maqu_2220* has broad substrate specificity. The difference in composition profile for the same *far* gene in different strains might be related to different growth medium and culturing conditions. Carbon chain lengths and degrees of saturation affect the physiochemical properties of fatty alcohols enabling different applications. By expressing different *far* genes and optimizing medium and fermentation conditions (e.g., different carbon sources), the carbon chain length of fatty alcohols may be tailored according to different substrate specificities of various *far* genes, and this could be explored in future work.

### Fatty alcohol production with glucose and xylose as substrate

**Fig. 7:**
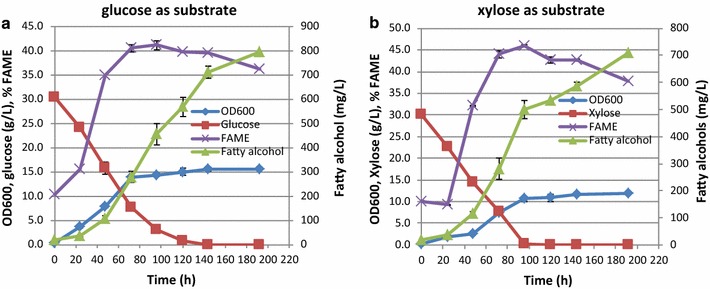
Cell growth, fatty alcohols, and lipid production by Ls[FAR] transformant 10 on mineral medium. **a** Glucose as substrate. **b** Xylose as substrate

An important characteristic of *L. starkeyi* is that it can utilize both glucose and xylose. The profile of fatty alcohol production by transformant 10 with glucose as substrate is depicted in Fig. 7a. Cell growth reached the stationary phase around 72 h even though the sugars were not completely consumed. The limited nitrogen content (0.5 g/L) for these cultures likely lead to a cessation of cell growth while still allowing fatty alcohol production. Lipid accumulation reached 40% within 72 h and slowly decreased thereafter, although glucose was still being consumed by the strain. It is noteworthy that fatty alcohols production increased as cultivation continued, reaching 735 mg/L at 144 h. At an extended fermentation, the fatty alcohols titer could reach up to about 800 mg/L at 192 h. After 72 h, it appears that the glucose consumption did not further contribute to cell growth or lipid synthesis, but fatty alcohols production continued at the same rate. Interestingly, once the glucose were exhausted at 120 h, the production of fatty alcohols continued but at a slower rate. This could be due to continued metabolic activity (e.g., de novo fatty alcohol production) or continued secretion of fatty alcohols that had built up inside of the cell previously. Clearly further experiments are warranted to understand the timing and rates of intracellular fatty alcohol production. Also of interest is the rapid use of xylose by *L. starkeyi*. Typically glucose is the preferred carbon source of any given organism, however *L. starkeyi* can utilize xylose as well as glucose.

The profile of fatty alcohol production by transformant 10 with xylose as substrate is similar to that using glucose as substrate, except that cell mass and the fatty alcohol titer were slightly lower on xylose than that on glucose (Fig. 7b). It was found that 587 mg/L fatty alcohols were produced at 144 h. With an extended incubation, the fatty alcohols titer could reach up to 710 mg/L at 192 h. We noted that the rate of xylose utilization is as fast as that of glucose, which is advantageous for utilizing biomass-derived sugars as feedstocks. The ability of *L. starkeyi* capable of producing high level of fatty alcohols from xylose makes it very attractive to use lignocellulosic biomass feedstocks. Although *R. toruloides* was shown to produce fatty alcohol from mixture of glucose and xylose, the levels are very low (220 mg/L); However, the rate of the fatty alcohol production in *L. starkeyi* is rather slow as compared to *R. toruloides.* [32].

The yields of fatty alcohols growing on glucose or xylose are summarized in Table 4. From these data, we note that the yields were low, which indicates that other steps in fatty alcohol synthesis pathway might be rate-limiting. Engineering other genes in fatty acid synthesis pathway, i.e., knocking out genes leading to TAG synthesis or overexpressing genes leading to more accumulation of fatty acyl-CoA might help to achieve high fatty alcohol titers.

**Table 4:** Yields of fatty alcohols on glucose or xylose as substrate (g/g glucose or xylose)

	Time (h)							
	0	24	48	72	96	120	144	192
Yield on glucose (g/g)	0.00	0.01	0.01	0.01	0.02	0.02	0.02	0.03
Yield on xylose (g/g)	0.00	0.00	0.01	0.01	0.02	0.02	0.02	0.02

**Fig. 8:**
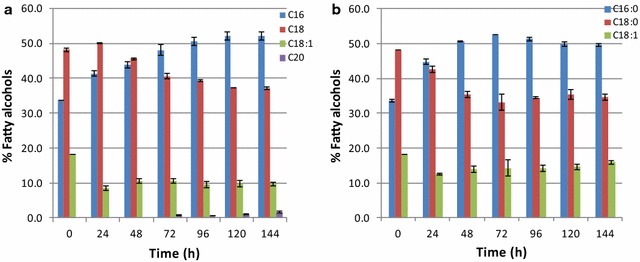
Pattern of fatty alcohol composition during fatty alcohol production process. **a** Glucose as substrate. **b** Xylose as substrate

During fermentations on glucose and xylose, the patterns of fatty alcohol composition changing with time were similar (Fig. 8). At early stage, i.e., 24 h, C18:0 fatty alcohol was preferentially produced. After 72 h, the percentage of C16:0 fatty alcohol increased, while C18:0 fatty alcohols dropped. In contrast, the percentage of C18:1 fatty alcohol remained nearly constant throughout the fermentation. It is noteworthy that, regardless of the change in the ratios of C16:0 and C18:0 fatty alcohols, the amounts of C16 and C18 fatty alcohols together accounted for about 85–90% of total fatty alcohols throughout the cultivation process.

### Distribution of fatty alcohols produced by *far*-expressing *L. starkeyi*

The distributions of the fatty alcohols produced by *far*-expressing *Y. lipolytica* and *L. starkeyi* were quite different. In *Y. lipolytica*, only 3% of the total was secreted, while in *L. starkeyi*, more fatty alcohols, at least 50% of the total was secreted from the cells into the medium. The partition of fatty alcohols in *L. starkeyi* is similar to that in *far*-expressing *R. toruloides* where 80% of fatty alcohols were secreted into culturing broth [32]. In the beginning, this striking difference in secretion led us think that there might be a threshold for fatty alcohols to secrete out instead of retaining intracellularly because the fatty alcohol titers in *L. starkeyi* and *R. toruloides* were much higher compared to that in *Y. lipolytica*. However, we got the same partition profile in low fatty alcohol producing *L. starkeyi* transformants (~200 mg/L fatty alcohols), i.e., more than half of the total fatty alcohols were secreted into the broth. This difference in fatty alcohol secretion titers for these strains could be related to the different permeabilities of cell membranes. Therefore, a structural and compositional analysis of the cell membranes might help elucidate the mechanism underlying the partition phenomena. Undoubtedly, the secretion of fatty alcohols could be beneficial for downstream product processing (e.g., separations), as well as for cell viability, considering the potential for fatty alcohols to be toxic.

## Conclusions

In summary, this study demonstrates fatty alcohol production by the oleaginous yeast, *Y. lipolytica* and *L. starkeyi*. By expressing the *far* gene from *M. aquaeolei* VT8 in both strains, 167 and 770 mg/L fatty alcohols were generated by *Y. lipolytica* and *L. starkeyi* growing on glucose, respectively. Furthermore, *Y. lipolytica* has been considered a model strain for the study of fatty acid synthesis in recent years. Our work demonstrates that, in addition to *Y. lipolytica*, *L. starkeyi* can also serve as a platform organism for production of fatty acid-derived biofuels and bioproducts via metabolic engineering. Our results show that *L. starkeyi far* gene transformant was able to produce fatty alcohols from both glucose and xylose, providing a basis for further engineering aimed at increasing fatty alcohol titer on cellulosic biomass feedstocks. To this end, more strategies are needed to enhance the fatty alcohol production, e.g., increase the copy number of the *far* gene. We believe that strain and process development can be combined to significantly contribute to the goal of producing scalable and cost-effective fatty alcohols from renewable biomass.

## Abbreviations

FAR: fatty acyl-CoA reductase
TAG: triacylglycerol
FAME: fatty acid methyl esters
DGA: diacylglycerol acyltransferase
